# Supplementary material for: CRPGAN: Learning image-to-image translation of two unpaired images by cross-attention mechanism and parallelization strategy
Source: PLoS One. 2023 Jan 6;18(1):e0280073. doi: 10.1371/journal.pone.0280073 (PMC9821492; doi:10.1371/journal.pone.0280073)
Supplement: Attachment — Submitted filename: comments on PONE-D-22-26766.pdf [file pone.0280073.s001.pdf]

This manuscript introduced the cross-attention mechanism and parallelization strategy into GAN for image-to-image translation of unpaired images. The idea is interesting and the result is basically satisfactory. However, some other problems in the manuscript are still concerned in the following:

1. In the experiments, the proposed method was compared with four methods, which are published in 2017, 2018, 2019, and 2020. In order to validate the effectivity more extensively, could the authors compare with more recent works?
2. Could the authors do ablation experiments on loss function?
3. The keywords are missing?
4. The organization of this manuscript should be added to the end of the introduction.
5. More recent works on image translation could be included, such as "DOI: 10.1109/TMM.2022.3147425", "DOI: 10.1016/j.isprsjprs.2021.07.007"…
6. Some references missed the fundamental information.
