# Supplementary material for: CRPGAN: Learning image-to-image translation of two unpaired images by cross-attention mechanism and parallelization strategy
Source: PLoS One. 2023 Jan 6;18(1):e0280073. doi: 10.1371/journal.pone.0280073 (PMC9821492; doi:10.1371/journal.pone.0280073)
Supplement: Attachment — Submitted filename: Response to Reviewers.docx [file pone.0280073.s002.docx]

Dear Editors and Reviewers:

Thank you for your letter and for the reviewers’comments concerning our manuscript entitled “CRPGAN: Learning image-to-image translation of two unpaired images by cross-attention mechanism and parallelization strategy” (ID: PONE-D-22-26766). Those comments are all valuable and very helpful for revising and improving our paper, as well as the important guiding significance to our researches. We have studied comments carefully and have made correction which we hope meet with approval. Revised portion aremarked in blue in the paper. The main corrections in the paper andthe responds to the reviewer’s comments are as flowing:

Responds to the reviewer’s comments:

Reviewer #1:

Response to comment: In the experiments, the proposed method was compared with four methods, which are published in 2017, 2018, 2019, and 2020. In order to validate the effectivity

more extensively, could the authors compare with more recent works?

Response: We added the image translation methods of the last two years as a baseline for qualitative and quantitative experiments with the methods in this paper, mainly DCLGAN, lrwGAN in 2021 and StyTr2, Qs-Attn in 2022.

DCLGAN bases on contrastive learning and a dual learning setting to infer an efficient mapping between unpaired data. lrwGAN solves the image translation task between two unaligned domains by importance re-weighted image selection. StyTr2 is a style transfer model using transformers as encoders. Qs-Attn design a query-selected attention (QS-Attn) module to ensure that the source image learns the target image features at the corresponding location for image translation. All of the above methods are typical of image translation and are similar to the data used in our method.

DCLGAN in our reference of [19]. lrwGAN in our reference of [23]. StyTr2 in our reference of [53]. Qs-Attn in our reference of [24].

Response to comment: Could the authors do ablation experiments on loss function?

Response: The loss function of the ablation experiment conducted in this paper is in Experiment in D, Ablation Study on the page 17.

We designed ablation experiments based on Summer↔Winter in order to verify the role of content loss, cyclic consistency loss, total variation loss and Cross-CBAM mechanism in one-shot image translation. We found that content loss preserves image content information, cyclic consistency loss prevents loss of image information, total variation loss prevents noise in images, and Cross-CBAM mechanism is used to generate finer style images.

Response to comment: The keywords are missing?

Response: We are very sorry that the Latex template for PLOS One cannot add keywords. But we have keywords, which are Unsupervised image-to-image translation, multi-scale training, progressive growth generator, parameter sharing, CRCBAM mechanism.

Response to comment:The organization of this manuscript should be added to the end of the

introduction.

Response: We have added the organization of the article and located it at the end of the introduction, in the red section on the page 4 . The organization of this manuscript is as follows:

The paper is structured as follows. Section 1 is a general introduction providing the necessary background and problems of unsupervised image translation. Section 2 provides current work related to style transfer, unsupervised image translation, and one-shot image translation. Section 3 provides the network structure, attention mechanism and loss function of the methods in this paper. Section 4 provides experimental setup, baseline methodology and evaluation metrics. Section 5 provides qualitative and quantitative experiments and the corresponding parametric and ablation studies. Section 6 provides a conclusion of our approach and future work.

Response to comment:More recent works on image translation could be included.

Response: We have added new image translation jobs, The references are as follows
[13]: Multi-View Consistent Generative Adversarial Networks for 3D-aware Image Synthesis, CVPR 2022

[14]: Bridge-GAN: Interpretable Representation Learning for Text-to-image Synthesis, TCSVT 2020
[15]: Dual Projection Generative Adversarial Networks for Conditional Image Generation, ICCV 2021
[16]: CM-GANs: Cross-modal Generative Adversarial Networks for Common Representation Learning, TOMM 2019

[19]: Dual Contrastive Learning for Unsupervised Image-to-Image Translation, CVPR 2021

[21]: Asynchronous generative adversarial network for asymmetric unpaired image-to-image translation. IEEE Transactions on Multimedia, 2022.

[22]: A deep translation (GAN) based change detection network for optical and SAR remote sensing images. ISPRS Journal of Photogrammetry and Remote Sensing, 2021
[23]: Unaligned Image-to-Image Translation by Learning to Reweight, ICCV 2021

[24]:QS-Attn: Query-Selected Attention for Contrastive Learning in I2I Translation, CVPR 2022

[53]: StyTr2: Image Style Transfer with Transformers, CVPR 2022

Response to comment: Some references missed the fundamental information.

Response: Thank you very much for your correction. We have carefully checked each referenceand revised it as follows: for the references cited with DOI, the URL prefix was removed and only the DOI number was retained.

Special thanks to you for your good comments.

Reviewer #2:

Response to comment: The proposed method refines the generated images by adding new convolution blocks continuously in different scales, and generates more refined style images with a new Cross-CBAM attention mechanism.

Response: Thank you for your affirmation and other very constructive suggestions. We will continue our efforts to delve deeper into issues in other areas.

Response to comment: The effectiveness of the proposed method should be further verified. In the original paper of comparison methods TuiGAN and SinGAN, the SIFID results are averaged across different general UI2I tasks, while comparison experiments are only conducted on 2 tasks (Summer-Winter, Horse-Zebra). The other datasets are not mentioned in the paper, such as OperaFace, Monet-Photo, GrumpifyCat, and Van Gogh-Photo. The settings of experiments should be identical for a fair comparison.
Response: Considering your suggestion, we have added photo→Van Gogh for qualitative and quantitative experiments of the baseline method. However, we did not add this experiment again due to the similarity between photo→Monet and photo→Van Gogh. The image translation between horse and zebra can represent the image translation between objects, therefore, in the object translation methods, such as OperaFace and GrumpifyCat, we did not add the remaining sets of comparison experiments, and only put the experimental results of the methods in this paper in the E, Object Transformation section of the final Result. Thank you again for your guidance and for making us understand the benefits of comparative experiments.

Response to comment: The latest comparison methods are published in 2020, and more recent SOTA methods, such as [a], [b], should be compared to further verify the effectiveness of the proposed method.

Response: We added the image translation methods of the last two years as a baseline for qualitative and quantitative experiments with the methods in this paper to further verify the effectiveness of the proposed method, mainly DCLGAN[a], lrwGAN[b] in 2021 and StyTr2, Qs-Attn in 2022.

DCLGAN bases on contrastive learning and a dual learning setting to infer an efficient mapping between unpaired data. lrwGAN solves the image translation task between two unaligned domains by importance re-weighted image selection. StyTr2 is a style transfer model using transformers as encoders. Qs-Attn design a query-selected attention (QS-Attn) module to ensure that the source image learns the target image features at the corresponding location for image translation. All of the above methods are typical of image translation and are similar to the data used in our method.

DCLGAN in our reference of [19]. lrwGAN in our reference of [23]. StyTr2 in our reference of [53]. Qs-Attn in our reference of [24].

Response to comment: There are many typos in the paper. For example, the number of references for method TSIT in table 2 should be [44], instead of [25]. And the name of the proposed method happens to be “ParGAN” in the last second paragraph of the Introduction, which should be “CRPGAN”. The authors may want to proofread the whole paper to carefully polish the writing.

Response: Thank you very much for your correction. We double-checked the index of the paper and the wrong words, and have revised the problems. Revise the following: correct the incorrect "ParGAN" to "CRPGAN", and correct the incorrect reference number [25] in TSIT to [52] after adding the reference.

.

Response to comment:The visualization results should be further refined. For example, the difference between columns (b), (c), and (d) is not clear enough to directly show the advantages of the proposed Cross-CBAM attention mechanism.

Response: Considering your suggestion, in Figure 5(a)(b)(c)(d) on the page 10, we have partially scaled up the experimental results of the Cross-CBAM attention mechanism, and we can find that the method(d) in this paper preserves more information. The translated images in b and c relatively blur at the edges and generate a large amount of erroneous blue information on the left side.

Response to comment: The end of each line should be aligned for a better outlook of the paper.

Response: Thank you for your corrections on the typography issue. We are very sorry that this is a requirement of the PLos One template.

Response to comment: More related works about “GAN”’s application on different tasks should be included to improve the completeness of the paper, such as [c], [d], [e], and [f].
Response: We have added new image translation jobs, such as [13], [14], [15], [16], [19], [21], [22], [23], [24] and [53] and used [15], [23], [24] and [53] as baseline methods.

[13]: Multi-View Consistent Generative Adversarial Networks for 3D-aware Image Synthesis, CVPR 2022

[14]: Bridge-GAN: Interpretable Representation Learning for Text-to-image Synthesis, TCSVT 2020
[15]: Dual Projection Generative Adversarial Networks for Conditional Image Generation, ICCV 2021
[16]: CM-GANs: Cross-modal Generative Adversarial Networks for Common Representation Learning, TOMM 2019

[19]: Dual Contrastive Learning for Unsupervised Image-to-Image Translation, CVPR 2021

[21]: Asynchronous generative adversarial network for asymmetric unpaired image-to-image translation. IEEE Transactions on Multimedia, 2022.

[22]: A deep translation (GAN) based change detection network for optical and SAR remote sensing images. ISPRS Journal of Photogrammetry and Remote Sensing, 2021
[23]: Unaligned Image-to-Image Translation by Learning to Reweight, ICCV 2021

[24]:QS-Attn: Query-Selected Attention for Contrastive Learning in I2I Translation, CVPR 2022

[53]: StyTr2: Image Style Transfer with Transformers, CVPR 2022

Special thanks to you for your good comments.

Other changes:

1. Removed the "B, Dataset in Experiment"

We tried our best to improve the manuscript and made some changes in the manuscript. These changes will not influence the content and framework of the paper. And here we did not list the changes but marked in red in revised paper.

We appreciate for Editors/Reviewers’ warm work earnestly, and hope that the correction will meet with approval.

Once again, thank you very much for your comments and suggestions.
